# Supplementary material for: Maternal Fructose Intake Causes Developmental Reprogramming of Hepatic Mitochondrial Catalytic Activity and Lipid Metabolism in Weanling and Young Adult Offspring
Source: Int J Mol Sci. 2022 Jan 17;23(2):999. doi: 10.3390/ijms23020999 (PMC8780605; doi:10.3390/ijms23020999)
Supplement: Supplementary file 1 [file ijms-23-00999-s001.zip › ijms-1507470-supplementary.pdf]

Supplementary Figure S1.

Adult Offspring DXA Scans

A

Control Male DXA-Month 4

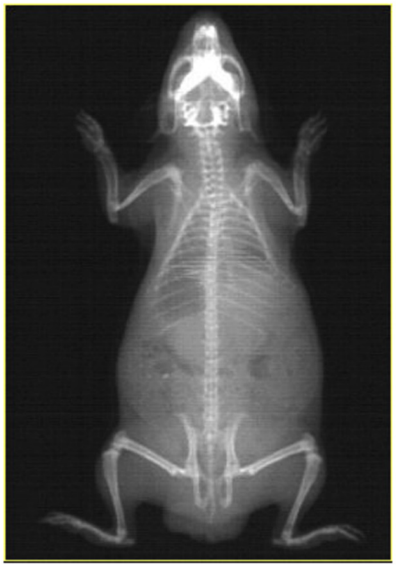

B

Fructose Male DXA-Month 4

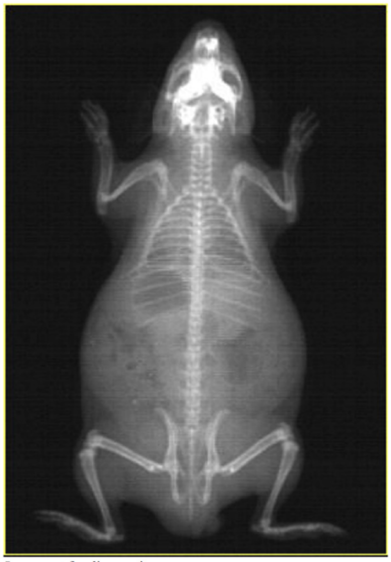

C

Control Female DXA-Month 4

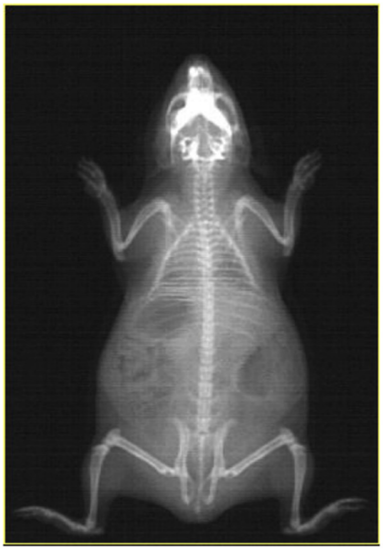

D

Fructose Female DXA-Month 4

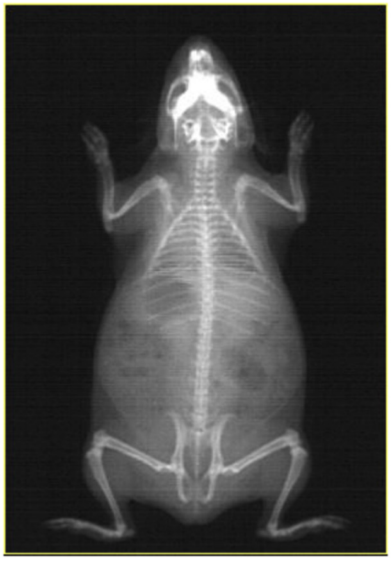

Supplementary Figure S2.

Weanling Hepatic Oil-Red-O Histology

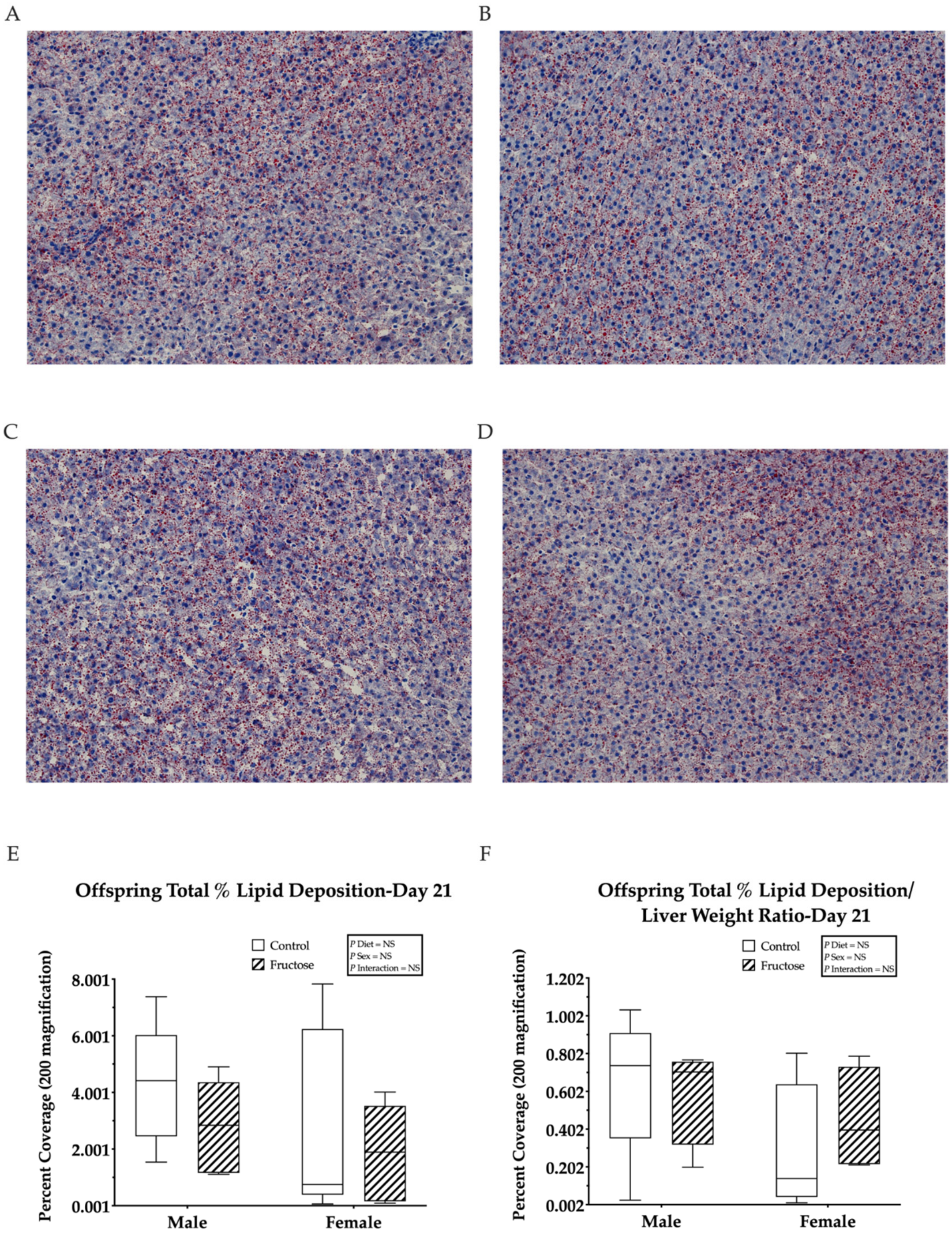

Supplementary Figure S3.

Adult Hepatic Oil-Red-O Histology

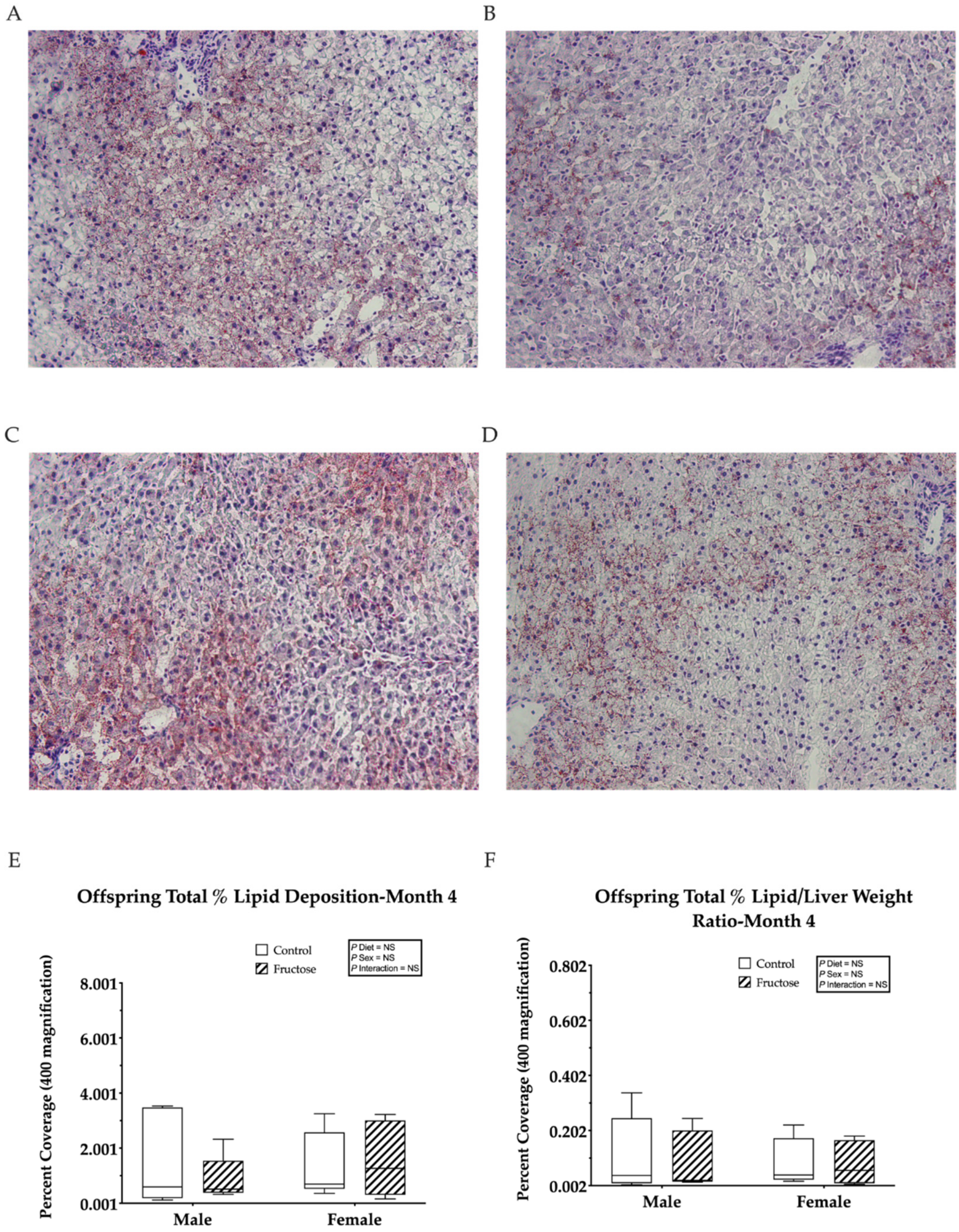

Supplementary Figure S4.

Adult Visceral Fat H&E Histology

A

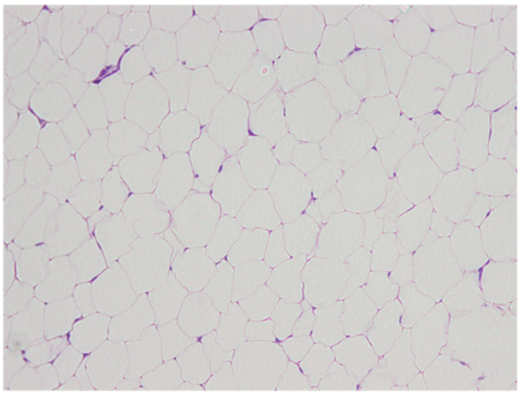

B

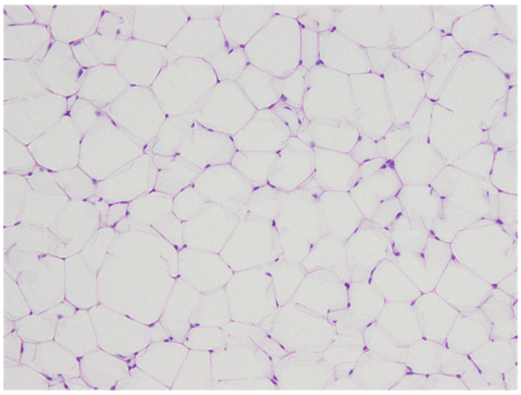

C

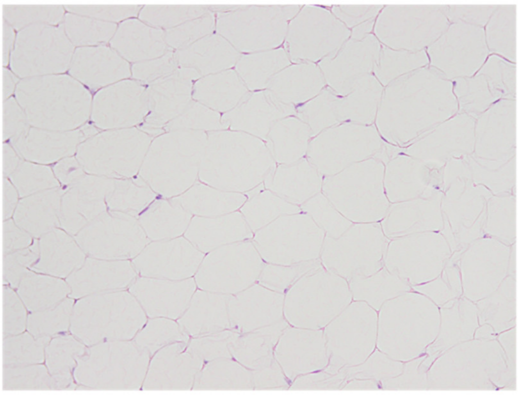

D

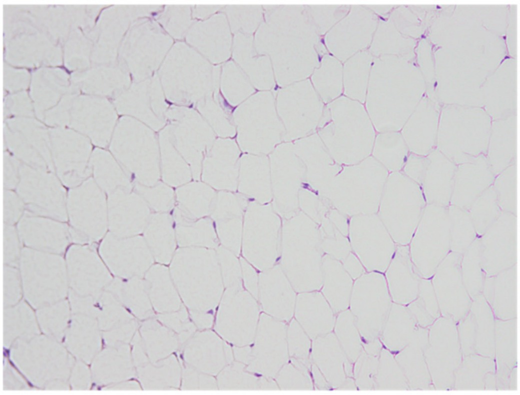

E

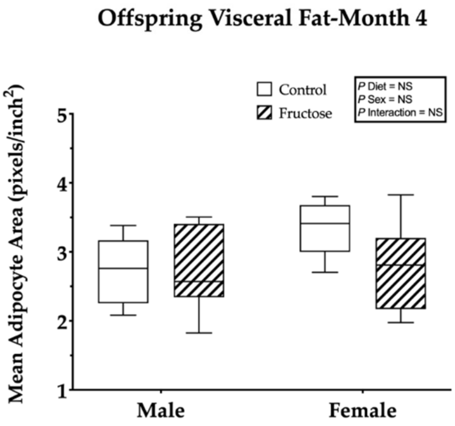

Supplementary Figure S5. Proteomics and ingenuity pathway analysis (IPA) reveals upregulation of pathways of oxidative phosphorylation and mitochondrial dysfunction in weanling (d21) FD male offspring.

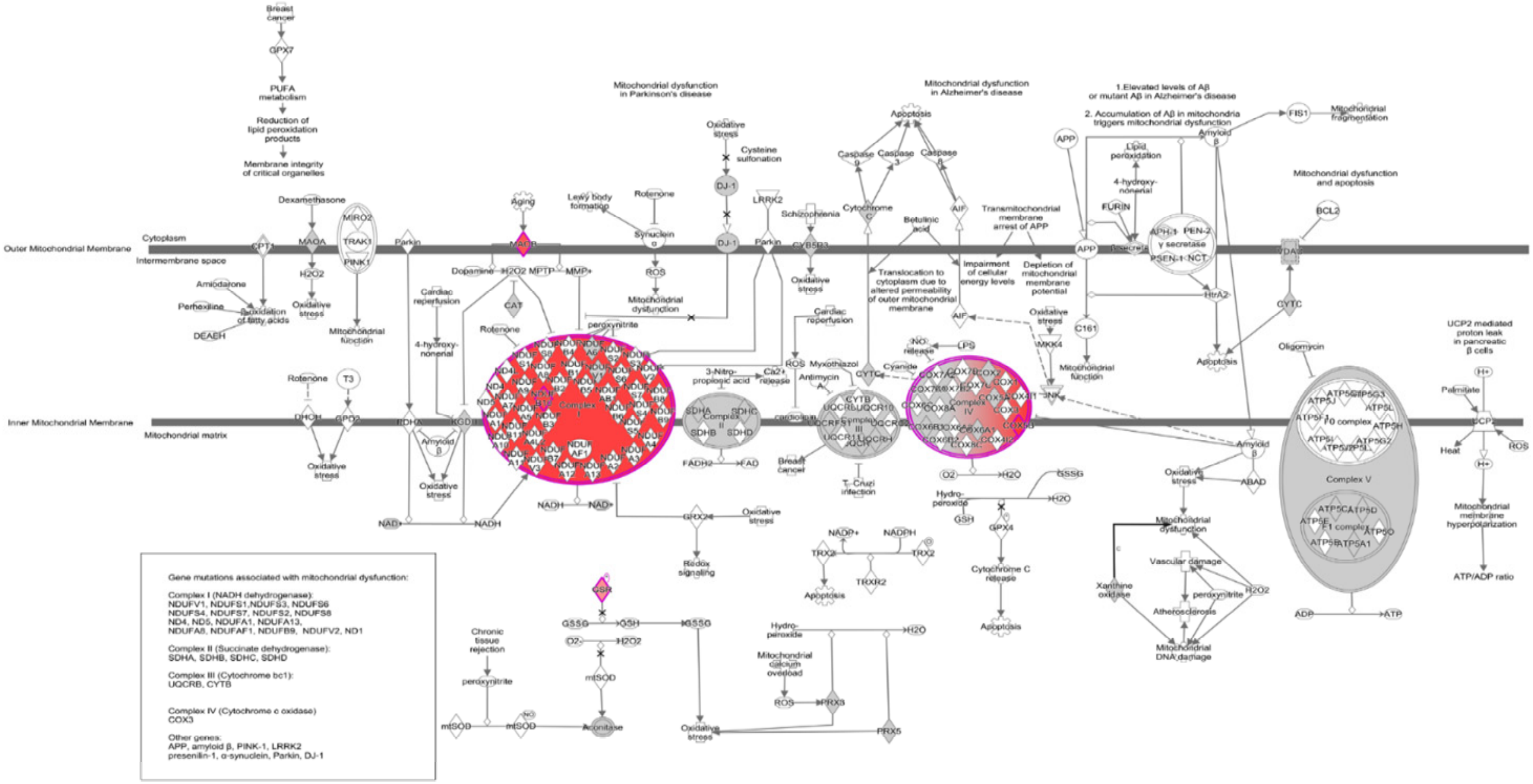

Supplementary Figure S6. Proteomics and ingenuity pathway analysis (IPA) reveals upregulation of NRF2-mediated oxidative stress response activity in weanling (d21) FD male offspring.

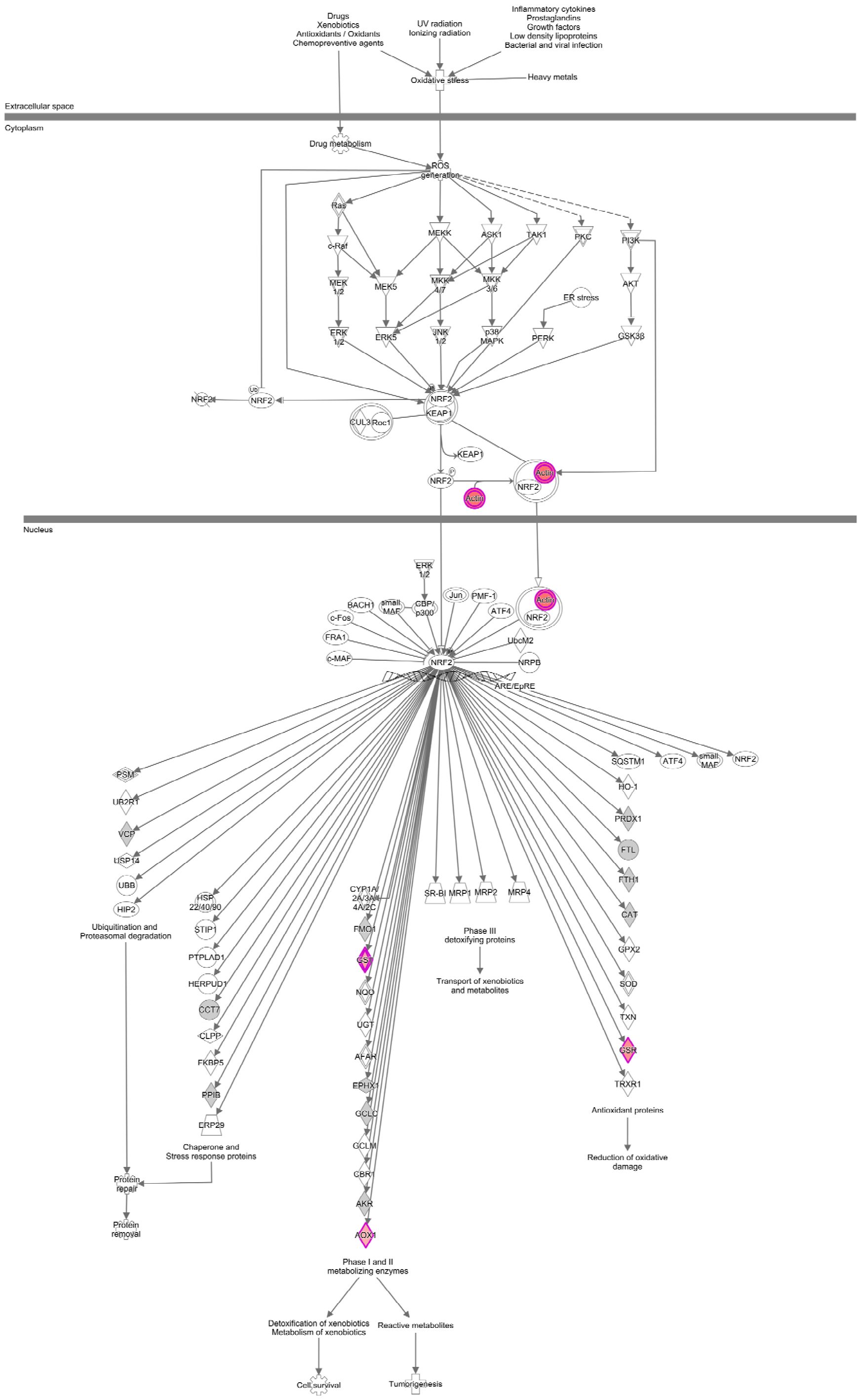

Supplementary Figure S7. Proteomics and ingenuity pathway analysis (IPA) reveals upregulation of fatty acid β-oxidation I pathway in weanling (d21) FD female offspring.

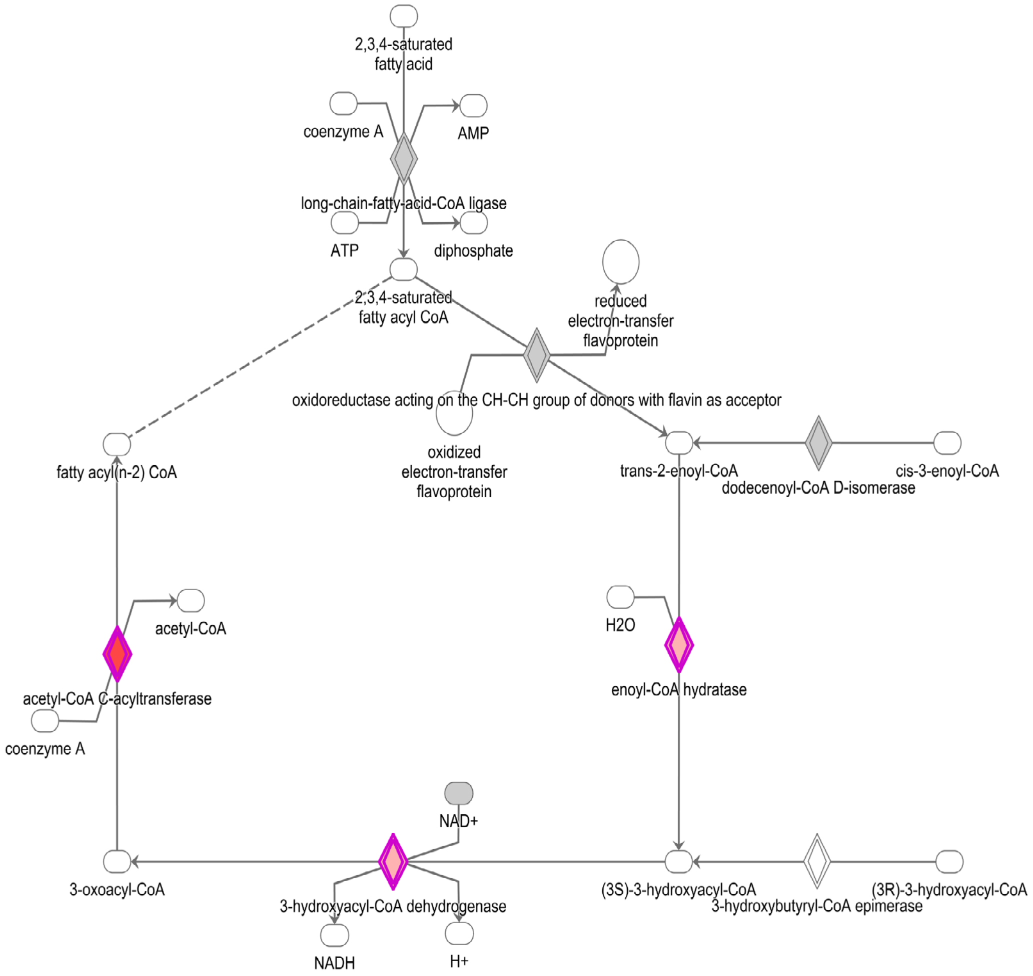

Supplementary Figure S8. Proteomics and ingenuity pathway analysis (IPA) reveals upregulation of mitochondrial dysfunction in adult (4M) FD male offspring.

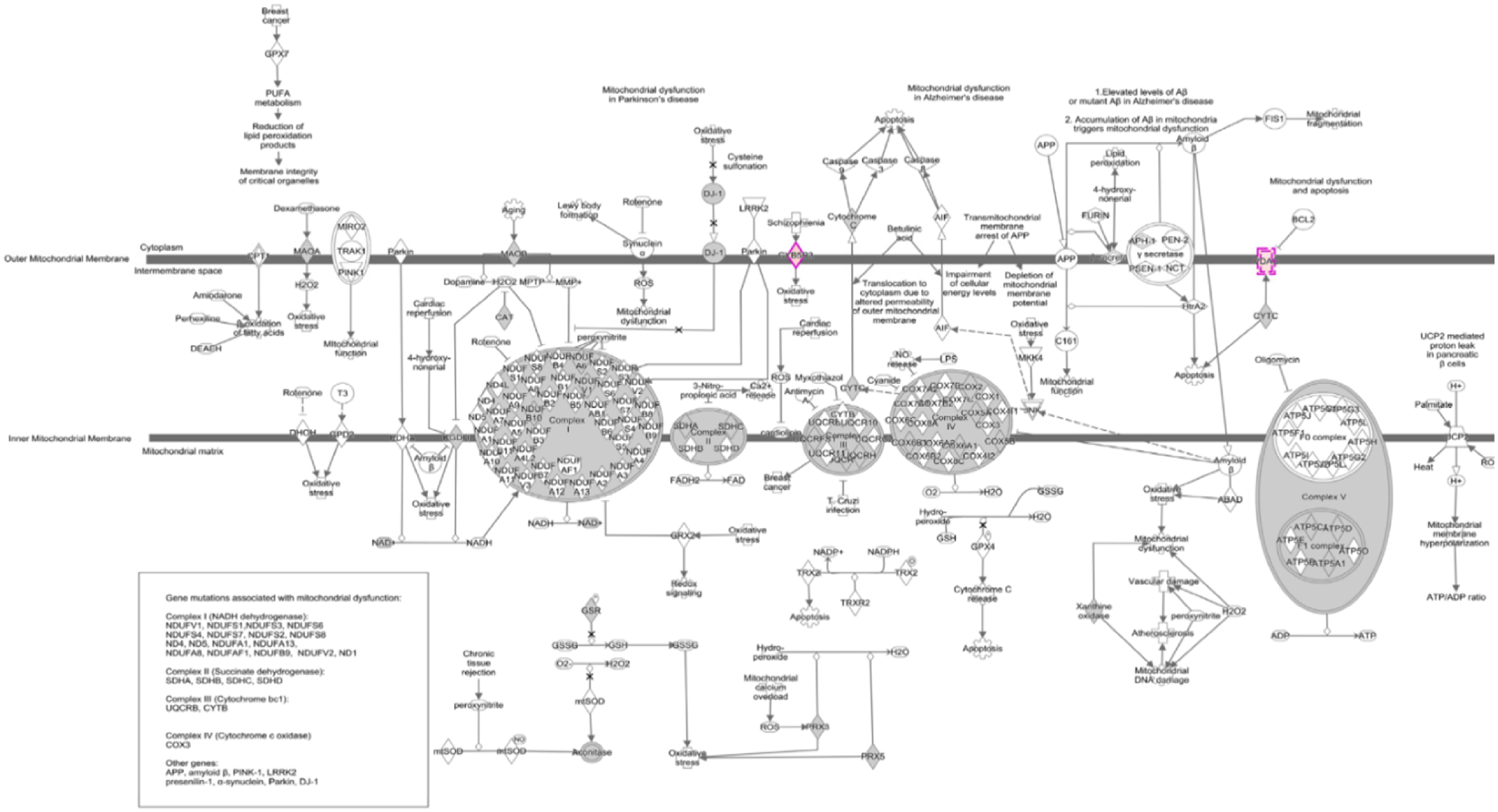

Supplementary Figure S9. Proteomics and ingenuity pathway analysis (IPA) reveals upregulation of pathways of fatty acid β-oxidation III, oxidative phosphorylation and mitochondrial dysfunction in adult (4M) FD female offspring.

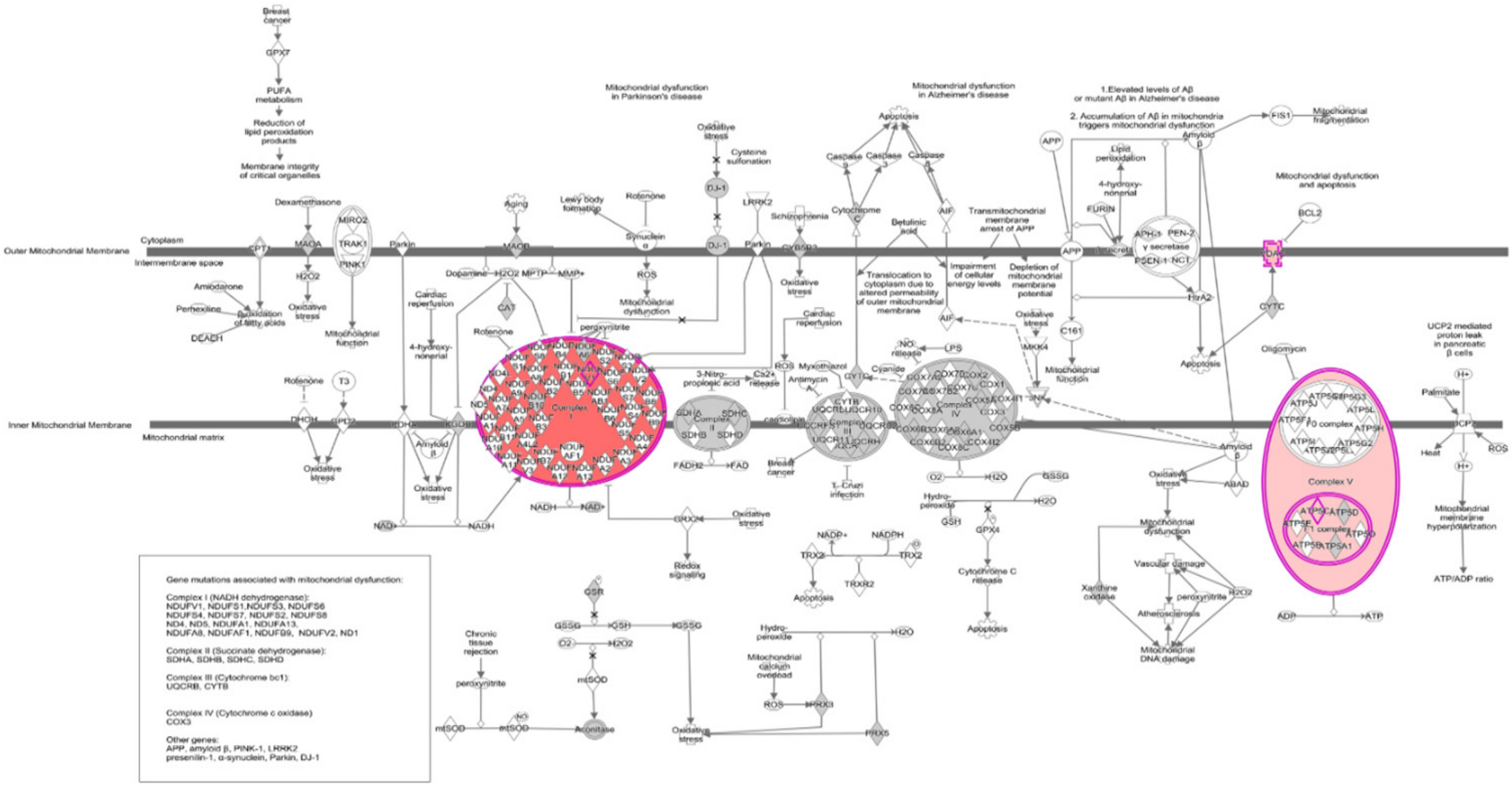

Supplementary Table S1.

Weanling and Adult Organ Weights

Offspring Organ Weights (g)	Sex	Day 21		4 Month	
		Control	Fructose	Control	Fructose
Liver	Male	6.36 ±0.17	5.14 ±0.17*	22.30 ±0.56*	23.73 ±0.56*
	Female	5.51 ±0.17*	4.73 ±0.17*,*	18.88 ±0.56	21.65 ±0.56*
Liver to Body Weight Ratio	Male	0.03 ±0.0004	0.02 ±0.0004	0.02 ±0.00	0.03 ±0.00*
	Female	0.02 ±0.0004	0.02 ±0.0004	0.02 ±0.00	0.03 ±0.00*
Visceral Fat	Male	0.95 ±0.87	0.30 ±0.87*	5.12 ±0.23	4.99 ±0.23
	Female	0.44 ±0.87*	0.22 ±0.87*,*	6.70 ±0.23*	5.62 ±0.23*
Visceral Fat to Body Weight Ratio	Male	0.004 ±0.00	0.002 ±0.00*	0.006 ±0.00	0.006 ±0.00*
	Female	0.002 ±0.00*	0.001 ±0.00*,*	0.01 ±0.00**	0.008 ±0.00*,**
Brain	Male	3.20 ±0.02	2.97 ±0.02*	4.05 ±0.02*	4.01 ±0.02*
	Female	3.08 ±0.02	3.02 ±0.02*	9.95 ±0.02	3.82 ±0.02
Brain to Body Weight Ratio	Male	0.015 ±0.00	0.017 ±0.00*	0.005 ±0.00	0.005 ±0.00
	Female	0.016 ±0.00	0.018 ±0.00*	0.006 ±0.00*	0.005 ±0.00*
Hippocampus	Male	0.14 ±0.00	0.12 ±0.00	0.15 ±0.00*	0.16 ±0.00*
	Female	0.12 ±0.00	0.13 ±0.00	0.15 ±0.00	0.12 ±0.00*
Cerebellum	Male	0.41 ±0.01	0.34 ±0.01*	0.51 ±0.02	0.57 ±0.02
	Female	0.30 ±0.01	0.39 ±0.01*	0.63 ±0.02	0.44 ±0.02*
Heart	Male	0.79 ±0.02	0.58 ±0.02**	2.24 ±0.05	2.36 ±0.05
	Female	0.71 ±0.02	0.56 ±0.02**	2.19 ±0.05	2.02 ±0.05
Heart to Body Weight Ratio	Male	0.0038 ±0.00	0.0033 ±0.00*	0.002 ±0.00	0.003 ±0.00
	Female	0.0037 ±0.00	0.0033 ±0.00*	0.003 ±0.00	0.003 ±0.00
Kidney	Male	1.69 ±0.03	1.38 ±0.03**	4.22 ±0.11**	4.64 ±0.11*,**
	Female	1.57 ±0.03*	1.34 ±0.03**,*	3.51 ±0.11	3.80 ±0.11*
Kidney to Body Weight Ratio	Male	0.007 ±0.00	0.007 ±0.00	0.005 ±0.00*	0.006 ±0.00*
	Female	0.007 ±0.00	0.008 ±0.00	0.005 ±0.00	0.005 ±0.00
Adrenal	Male	0.06 ±0.03	0.06 ±0.03	0.29 ±0.00	0.29 ±0.00
	Female	0.09 ±0.03	0.18 ±0.03	0.32 ±0.00	0.32 ±0.00
Subcutaneous Fat	Male	1.93 ±0.09	1.05 ±0.09**	7.76 ±0.48	6.62 ±0.48
	Female	1.72 ±0.09	1.17 ±0.09**	8.08 ±0.48*	10.55 ±0.48*,*

Supplementary Table S2.

Adult OGTT Glucose, Insulin and Matsuda-ISI

Adult OGTT Response	Dietary Group	Males	P-value	Females	P-value
		Blood Glucose (mmol/L)			
**Baseline**	Control	6.94 ± 0.21	0.78	6.56 ± 0.14	0.10
	Fructose	6.83 ± 0.29		6.93 ± 0.14	
**30mins**	Control	13.68 ± 0.93	0.51	11.18 ± 0.75	0.68
	Fructose	12.80 ± 0.92		11.63 ± 0.77	
**45mins**	Control	15.00 ± 1.40	0.95	12.50 ± 0.80	0.53
	Fructose	15.10 ± 1.10		11.66 ± 1.00	
**60mins**	Control	14.41 ± 1.20	0.63	11.64 ± 0.88	0.41
	Fructose	15.23 ± 1.14		10.80 ± 0.51	
**75mins**	Control	13.11 ± 1.33	0.57	11.06 ± 0.64	0.07
	Fructose	14.11 ± 1.07		9.60 ± 0.35	
**90mins**	Control	11.33 ± 1.10	0.73	8.68 ± 0.39	0.62
	Fructose	11.93 ± 1.35		8.38 ± 0.41	
**120mins**	Control	7.78 ± 0.42	0.98	6.94 ± 0.26	0.88
	Fructose	7.80 ± 0.78		7.00 ± 0.31	
**180mins**	Control	6.50 ± 0.10	1.00	6.30 ± 0.22	0.20
	Fructose	6.50 ± 0.09		6.86 ± 0.35	
		Plasma Insulin (μU/ml)			
**Baseline**	Control	2.13 ± 0.41	0.62	2.15 ± 0.45	0.90
	Fructose	1.85 ± 0.37		2.23 ± 0.49	
**30min**	Control	2.02 ± 0.33	0.22	2.35 ± 0.41	0.45
	Fructose	1.53 ± 0.19		1.84 ± 0.48	
**45min**	Control	1.88 ± 0.31	0.19	2.54 ± 0.33	0.17
	Fructose	1.40 ± 0.13		1.77 ± 0.40	
**60mins**	Control	1.74 ± 0.38	0.56	2.26 ± 0.32	0.20
	Fructose	1.46 ± 0.26		1.63 ± 0.33	
**75mins**	Control	1.38 ± 0.15	0.82	2.35 ± 0.45	0.26
	Fructose	1.44 ± 0.19		1.66 ± 0.36	
**90mins**	Control	1.71 ± 0.27	0.39	2.44 ± 0.39	0.13
	Fructose	1.40 ± 0.20		1.58 ± 0.87	
**120mins**	Control	2.18 ± 0.30	0.07	2.40 ± 0.51	0.32
	Fructose	1.44 ± 0.19		1.73 ± 0.39	
**180mins**	Control	1.98 ± 0.19	0.24	2.38 ± 0.39	0.33
	Fructose	1.61 ± 0.23		1.80 ± 0.53	
		Matsuda-ISI			
	Control	3.38 ± 0.50	0.33	3.61 ± 0.67	0.50
	Fructose	4.11 ± 0.51		4.35 ± 0.86	

Supplementary Table S3.

FD Weanling and Adult Hepatic Proteome and Mitochondrial Pathway Signaling

Signaling Pathways	Protein Names	Protein Symbol	*-log₁₀P*-value				Fold Change				*P*-value (t-test)			
			21d Male	21d Female	4M Male	4M Female	21d Male	21d Female	4M Male	4M Female	21d Male	21d Female	4M Male	4M Female
**Mitochondrial Dysfunction**			3.83		1.58	2.06								
	cytochrome c oxidase subunit 5B	COX5B	-	-	-	-	3	-	-	-	0.001	-	-	-
	glutathione-disulfide reductase	GSR	-	-	-	-	1.6	-	-	-	0.007	-	-	-
	monoamine oxidase B	MAOB	-	-	-	-	2.5	-	-	-	0.001	-	-	-
	NADH:ubiquinone oxidoreductase subunit B10	NDUFB10	-	-	-	-	2.8	-	-	-	0.003	-	-	-
	cytochrome b5 reductase 3	CYB5R3	-	-	-	-	-	-	1.8	-	-	-	0.05	-
	voltage dependent anion channel 1	VDAC1	-	-	-	-	-	-	1.2	-	-	-	0.03	-
	ATP synthase F1 subunit gamma	ATP5F1C	-	-	-	-	-	-	-	1.3	-	-	-	0.04
	NADH:ubiquinone oxidoreductase core subunit V1	NDUFV1	-	-	-	-	-	-	-	3.6	-	-	-	0.04
	voltage dependent anion channel 2	VDAC2	-	-	-	-	-	-	-	1.5	-	-	-	0.01
**Oxidative Phosphorylation**			1.9			1.53								
	cytochrome c oxidase subunit 5B	COX5B	-	-	-	-	3	-	-	-	0.001	-	-	-
	NADH:ubiquinone oxidoreductase subunit B10	NDUFB10	-	-	-	-	2.8	-	-	-	0.003	-	-	-
	ATP synthase F1 subunit gamma	ATP5F1C	-	-	-	-	-	-	-	1.3	-	-	-	0.04
	NADH:ubiquinone oxidoreductase core subunit V1	NDUFV1	-	-	-	-	-	--	-	3.6	-	-	-	0.04
**NRF2-mediated Oxidative Stress Response**			3.82	3.2										
	actin gamma 2, smooth muscle	ACTG2	-	-	-	-	2.1	-	-	-	0.03	-	-	-
	aldehyde oxidase 1	AOX1	-	-	-	-	1.2	-	-	-	0.03	-	-	-
	glutathione-disulfide reductase	GSR	-	-	-	-	1.6	-	-	-	0.007	-	-	-
	glutathione S-transferase alpha 1	GSTA1	-	-	-	-	1.3	-	-	-	0.008	-	-	-
	chaperonin containing TCP1 subunit 7	CCT7	-	1.3	-	-	-	-	-	-	-	0.05	-	-
	peptidylprolyl isomerase B	PPIB	-	1.7	-	-	-	-	-	-	-	0.025	-	-
	peroxiredoxin 1	PRDX1	-	2	-	-	-	-	-	-	-	0.04	-	-
**fatty acid β-oxidation I**				3.12										
	hydroxysteroid 17-beta dehydrogenase 4	HSD17B4	-	1.2	-	-	-	-	-	-	-	0.01	-	-

Signaling Pathways	Protein Names	Protein Symbol	$-log_{10}P$-value				Fold Change				*P*-value (t-test)			
			21d Male	21d Female	4M Male	4M Female	21d Male	21d Female	4M Male	4M Female	21d Male	21d Female	4M Male	4M Female
sterol carrier protein 2		SCP2	-	2.9	-	-	-	-	-	-	-	0.008	-	-
**fatty acid β-oxidation III**						1.87								
enoyl-CoA hydratase and 3-hydroxyacyl CoA dehydrogenase		EHHADH	-	-	-	-	-	-	-	1.1	-	-	-	0.01

Supplementary Figure Legends

Supplementary Figure S1. Adult offspring DXA scan images at month 4. CD males (n=6); FD males (n=9); CD females (n=7) and FD females (n=6). **A)** Represents CD male DXA scan image. **B)** Represents FD male DXA scan image. **C)** Represents CD female DXA scan image. **D)** Represents FD female DXA scan image.

Supplementary Figure S2. Weanling offspring hepatic lipid deposition example images of d21 offspring. All images are of 6μm thick sections taken from frozen d21 liver tissue, stained with oil-red-o and photographed at 200x magnification. Lipid droplets (red) among hepatocytes (light blue) and hepatocyte nuclei (dark blue). **A)** Represents image of CDM offspring lipid deposition at d21. **B)** Represents image of FDM offspring lipid deposition at d21. **C)** Represents image of CDF offspring lipid deposition at d21. **D)** Represents image of FDF offspring lipid deposition at d21. Weanling offspring hepatic total % lipid deposition and total % lipid deposition to liver weight ratio. CD males (n=6; FD males (n=5); CD females (n=5) and FD females (n=6). **E)** Represents offspring hepatic total % lipid deposition at d21. **F)** Represents offspring liver weight to total % lipid deposition ratio at d21. All data were analysed using a 2x2 factorial design with diet*sex*interaction include as factors (general analysis of variance) using IBM SPSS statistics 25. Data presented as group mean ± SEM.

Supplementary Figure S3. Adult Offspring hepatic lipid deposition example images of 4M offspring. CD males (n=7); FD males (n=9); CD females (n=7) and FD females (n=6). All images are of 6μm thick sections taken from frozen 4M liver tissue, stained with oil-red-o and photographed at 400x magnification. Lipid droplets (red) among hepatocytes (light blue) and hepatocyte nuclei (dark blue). **A)** Represents image of CDM offspring lipid deposition at 4M. **B)** Represents image of FDM offspring lipid deposition at 4M. **C)** Represents image of CDF offspring lipid deposition at 4M. **D)** Represents image of FDF offspring lipid deposition at 4M. Adult offspring hepatic total % lipid deposition and total % lipid deposition to liver weight ratio. CD males (n=7); FD males (n=9); CD females (n=7) and FD females (n=6). **E)** Represents offspring 400 magnification hepatic total % lipid deposition at month 4. **F)** Represents offspring 400 magnification liver weight to total % lipid deposition ratio at month 4. All data were analysed using a 2x2 factorial design with diet*sex*interaction included as factors (general analysis of variance) using IBM SPSS statistics 25. Data presented as group mean ±SEM.

Supplementary Figure S4. Adult Offspring visceral fat H&E example images of 4M offspring. CD males (n=7); FD males (n=7); CD females (n=6) and FD females (n=6). All images are of 4μm thick sections taken from fixed 4M visceral fat tissue, stained with H&E and photographed at 200x magnification. **A)** Represents image of CDM offspring visceral fat morphology at 4M. **B)** Represents image of FDM offspring visceral fat morphology at 4M. **C)** Represents image of CDF offspring visceral fat morphology at 4M. **D)** Represents image of FDF offspring visceral fat morphology at 4M. **E)** Adult Offspring visceral fat morphology. CD males (n=7); FD males (n=7); CD females (n=6) and FD females (n=6). All data were analysed using a 2x2 factorial design with diet*sex*interaction included as factors (general analysis of variance) using IBM SPSS statistics 25. Data presented as group mean ±SEM.

Supplementary Figure S5. Proteomics and ingenuity pathway analysis (IPA) reveals upregulation of pathways of oxidative phosphorylation and mitochondrial dysfunction in weanling (d21) FD male offspring. CD males (n=5) and FD males (n =5). Figure represents most significantly affected pathways ($-\log_{10}$ P-value =1.90 for oxidative phosphorylation and $-\log_{10}$ P-value =3.83 for mitochondrial dysfunction) based on increased protein expression and directionally theorised pathway activation in FD males compared to CD males. Red indicates up-regulation and grey

indicates ineligible prediction. The P-values are calculated using IPA's right-tailed fisher's exact test and $-\log_{10}$ P-values to represent significance.

Supplementary Figure S6. Proteomics and ingenuity pathway analysis (IPA) reveals upregulation of NRF2-mediated oxidative stress response activity in weanling (d21) FD male offspring. CD males (n=5) and FD males (n=5). Figure represents most significantly affected signaling pathways ($-\log_{10}$ P-value =3.82) based on increased protein expression and directionally theorised pathway activation in FD males compared to CD males. Red indicates up-regulation and grey indicates ineligible prediction. The P-values are calculated using IPA's right-tailed fisher's exact test and $-\log_{10}$ P-values to represent significance.

Supplementary Figure S7. Proteomics and ingenuity pathway analysis (IPA) reveals upregulation of fatty acid β-oxidation I pathway in weanling (d21) FD female offspring. CD females (n=5) and FD females (n=5). Figure represents most significantly affected pathways ($-\log_{10}$ P-value =3.12) based on increased protein expression and directionally theorised pathway activation in FD females compared to CD female. Red indicates up-regulation and grey indicates ineligible prediction. The P-values are calculated using IPA's right-tailed fisher's exact test and $-\log_{10}$ P-values to represent significance.

Supplementary Figure S8. Proteomics and ingenuity pathway analysis (IPA) reveals upregulation of mitochondrial dysfunction in adult (4M) FD male offspring. CD (n=5) and FD males (n=5). Figure represents most significantly affected pathways ($-\log_{10}$ P-value =1.58) based on increased protein expression and directionally theorised pathway activation in FD males compared to CD males. Red indicates up-regulation and grey indicates ineligible prediction. The P-values are calculated using IPA's right-tailed fisher's exact test and $-\log_{10}$ P-values to represent significance.

Supplementary Figure S9. Proteomics and ingenuity pathway analysis (IPA) reveals upregulation of pathways of fatty acid β-oxidation III, oxidative phosphorylation and mitochondrial dysfunction in adult (4M) FD female offspring. CD females (n=5) and FD females (n=5). Data represents most significantly affected signaling pathways ($-\log_{10}$ P-value =1.87 fatty acid β-oxidation III, $-\log_{10}$ P-value =1.53 for oxidative phosphorylation and $-\log_{10}$ P-value =2.06 for mitochondrial dysfunction) based on increased expression, directionally inferred pathway activation and association of hepatic proteins with predicted pathway activation or inhibition of analysed hepatic proteins with alterations in FD females compared to CD females. Red indicates up-regulation and grey indicates ineligible prediction. The P-values are calculated using IPA's right-tailed fisher's exact test and $-\log_{10}$ P-values to represent significance.

Supplementary Table Legend

Supplementary Table S1. Weanling (d21) and Adult (4M) organ weights. D21 offspring CD males (n=7); FD males (n=7); CD females (n=7) and FD females (n=7). 4M offspring CD males (n=7); FD males (n=9); CD females (n=7) and FD females (n=6). All data were analysed using a 2x2 factorial design with diet*sex*interaction included as factors (general analysis of variance) using IBM SPSS statistics 25. Data presented as group mean ±SEM.* denotes significance of P-value <0.05; **denotes significance of P-value <0.0001.

Supplementary Table S2. Adult offspring OGTT Glucose, Insulin and Matsuda-ISI. Adult offspring OGTT Glucose, Insulin and Matsuda-ISI. CD males (n=6); FD males (n=6); CD females (n=6) and FD females (n=6). All data were analysed using an independent T-test using IBM SPSS statistics 25. Data presented as group mean ±SEM.

Supplementary Table S3. FD weanling and adult offspring hepatic proteome and mitochondrial signaling pathways and associated proteins. Table represents signaling pathways 21d FD males compared to 21d CD males Control males (n=5) and fructose males (n=5), 21d FD females compared to 21d CD females Control females (n=4) and fructose females (n=5), 4M FD males compared to 4M CD males Control males (n=5) and fructose males (n=5) and 4M FD females compared to 4M CD females Control females (n=5) and fructose females (n=5). All data were analysed using Ingenuity Pathway Analysis (IPA). The P-values are calculated using IPA's right-tailed Fisher's exact test with -log10 P-values to represent significance of pathological endpoint and $P<0.05$ to represent significance of specific protein expression upregulated within the pathological endpoint.
